# 10-year follow-up of congenital cytomegalovirus infection complicated with severe neurological findings in infancy: a case report

**DOI:** 10.1186/s12887-018-1348-8

**Published:** 2018-11-23

**Authors:** Eisuke Suganuma, Akira Oka, Hideaki Sakata, Nodoka Adachi, Satoshi Asanuma, Eiji Oguma, Akira Yamaguchi, Mihoko Furuichi, Yoji Uejima, Satoshi Sato, Tadamasa Takano, Yutaka Kawano, Risa Tanaka, Takashi Arai, Tsutomu Oh-Ishi

**Affiliations:** 1 Division of Infectious Diseases and Immunology, Saitama Children’s Medical Center, 1-2 Shintoshin, Chuou-ku, Saitama-shi, Saitama 330-8777 Japan; 2 Department of Paediatrics, The University of Tokyo, Tokyo, Japan; 3 Division of Otorhinolaryngology, Kawagoe Otology Institute, Saitama, Japan; 4 Division of Otolaryngology, Saitama Children’s Medical Center, Saitama, Japan; 5 Division of Radiology, Saitama Children’s Medical Center, Saitama, Japan; 6 Department of Radiological Technology, Saitama Children’s Medical Center, Saitama, Japan; 7 Department of Pediatrics, Saitama Medical Center, Saitama Medical University, Saitama, Japan; 8 MicroSKY Lab, Inc., Tokyo, Japan; 9 The Medical and Nursing Institution of Akitsu Ryoiku-En for Children/Adults with Severe Motor and Intellectual Disabilities, Tokyo, Japan

**Keywords:** Cytomegalovirus, White matter abnormality, Sensorineural hearing loss, Ganciclovir, Neurodevelopment

## Abstract

**Background:**

Congenital cytomegalovirus (cCMV) infection leads to sensorineural hearing loss (SNHL) and neurodevelopmental delays. However, the long-term outcomes of cCMV infection with severe neurological manifestations in infancy remain unclear.

**Case presentation:**

The patient was a one-month-old girl visited owing to abnormalities in neonatal hearing screening. Central nervous system involvement including intracranial calcification and extensive white matter abnormalities was identified. Right SNHL (50 dB) was detected by auditory brain response (ABR) testing. The cause of her hearing loss was determined to be cCMV infection by polymerase chain reaction (PCR) using a dried blood spot. At 1.5 months of age, the patient was treated with intravenous ganciclovir (GCV) for 5 weeks followed by oral valganciclovir (VGCV) for an additional 6 weeks. Cytomegalovirus (CMV) loads in her urine continued to be detected until she was 10 years old. Fortunately, during this time, her right hearing loss did not deteriorate, and her left hearing remained normal. Furthermore, the extensive abnormal areas of white matter observed at 1 month of age mostly disappeared by the time the patient was 9 years old. Her neurodevelopmental score was normal, and motor milestones were not delayed as of 10 years of age.

**Conclusions:**

Here, we report the 10-year follow-up of a patient with cCMV who showed normal neurodevelopment, no progression of hearing loss, and ameliorating magnetic resonance imaging (MRI) findings, despite having various complications and severe neurological findings during infancy.

**Electronic supplementary material:**

The online version of this article (10.1186/s12887-018-1348-8) contains supplementary material, which is available to authorized users.

## Background

Cytomegalovirus (CMV) is a major cause of congenital infection and is one component of TORCH syndrome (toxoplasma, rubella, CMV infection, herpes simplex, and other agents) [1]. Approximately 10% of neonates with congenital CMV (cCMV) infection have symptomatic manifestations at birth, such as intrauterine growth retardation, hepatomegaly, jaundice, thrombocytopenia, blueberry muffin rash, microcephaly, and intracranial calcification, which can lead to neurodevelopmental complications including mental retardation and sensorineural hearing loss (SNHL). Approximately 70–80% of infants who are symptomatic at birth develop late complications that may include late-onset hearing loss, intellectual disabilities, balance disturbances, or psychomotor retardation [2–4].

However, the long-term outcomes of neurodevelopment and SNHL in children with cCMV infection have not been fully elucidated. Herein, we report the 10-year follow-up of a cCMV patient who exhibited normal neurodevelopment following antiviral drug administration despite having severe neurological findings during infancy.

## Case presentation

A female neonate was born to a healthy mother after normal labor at 40 weeks of gestation. Her birth weight was 3668 g (98th percentile), her height was 52 cm (93rd percentile), and her head circumference was 33.5 cm (53rd percentile). Her Apgar scores were 8 and 9 at one and 5 minutes, respectively. At the 30th week of gestation, her mother had an influenza A virus infection confirmed by an immunochromatographic kit using a monoclonal antibody against influenza A virus. There were no abnormal physiological findings at birth.

She was referred for newborn hearing screening of her right ear with automated auditory brainstem response (AABR) testing. At 1 month of age, she was referred to the otolaryngology department of our hospital. Right hearing loss (50 dB) was detected by auditory brainstem response (ABR) testing, whereas the left ear was normal (20 dB).

She was diagnosed with cCMV infection by a quantitative real-time polymerase chain reaction (PCR) assay using preserved dried blood spots on filter paper for newborn congenital metabolic disorder mass-screening. CMV DNA was also detected in urine (2.2 × 10^6^ copies/mL) and peripheral blood mononuclear cells (PBMCs) (6 copies/μg DNA) at 1 month of age, although no CMV DNA was detected in serum.

**Fig. 1:**
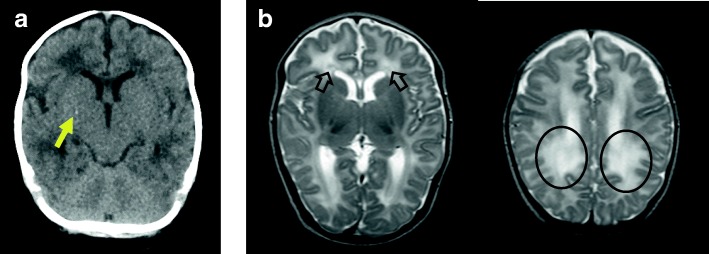
CT and MRI findings. **a** Head CT scan at 1.5 months of age. The arrow indicates intracranial calcification. **b** Axial T2-weighted brain MRI at 1.5 months of age showing diffuse abnormalities of the white matter in the parietal, occipital, and frontal lobes (circle) as well as periventricular cysts (arrows) around the posterior horn of the bilateral lateral ventricles. No polymicrogyria, lissencephaly, or ventriculomegaly is observed

Intracranial calcification was observed on head computed tomography (CT) (Fig. 1,a arrow). Furthermore, T2-weighted brain magnetic resonance imaging (MRI) at 1.5 months of age (Fig. 1b) showed diffuse, abnormal, high-signal areas of white matter in the parietal and occipital lobes (round circles) as well as bilateral periventricular cysts (arrows) around the anterior horn of the lateral ventricle. There was no evidence of ventricular enlargement, polymicrogyria, or microcephaly. Chorioretinitis was ruled out by pediatric ophthalmologic examination.

We informed her parents of the risks and benefits of antiviral drugs and started treatment with their consent, as approved by Institutional Review Board (IRB) of Saitama Children’s Medical Center. A dosage of 6 mg/kg twice daily of intravenous ganciclovir (GCV) was started to prevent progression of hearing loss. Because the patient’s liver enzymes were elevated (aspartate aminotransferase, AST: 339 IU/L, alanine aminotransferase, ALT: 361 IU/L) on the 18th day after the initiation of GCV treatment, GCV was temporarily discontinued. After her liver enzymes decreased, GCV was resumed at a half dose on the 25th day and continued until the 43rd day without additional adverse effects.

**Fig. 2:**
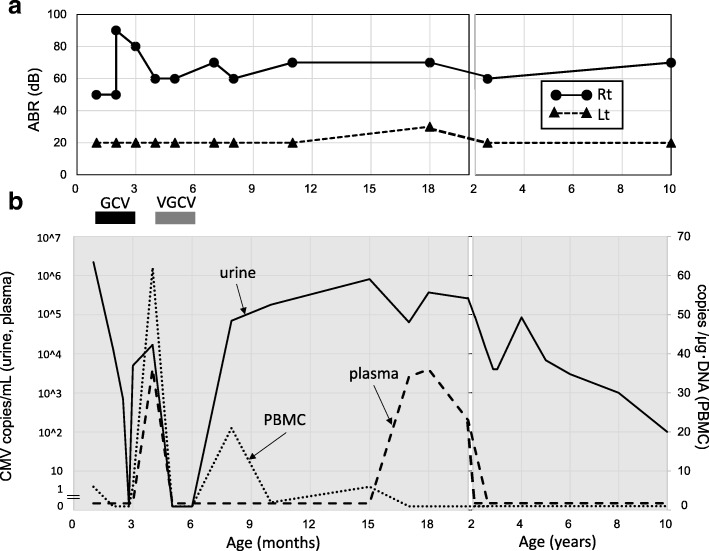
Hearing testing and CMV DNA copy numbers. **a** Time-course of hearing test results. **b** CMV DNA copy numbers. PBMC: peripheral blood mononuclear cell, GCV: ganciclovir, VGCV: valganciclovir

Although the urine CMV DNA copy number decreased from 2.2 × 10^6^ copies/mL at the beginning of treatment to 0/mL during GCV treatment, it rebounded to 5.0 × 10^3^ copies/mL 6 weeks later. Therefore, oral antiviral valganciclovir (VGCV) at 11 mg/kg twice daily was started and continued for an additional 6 weeks. Two months after completion of VGCV treatment, the urine CMV DNA copy number increased to 7.0 × 10^4^ copies/mL, which was similar to the copy number before treatment (1.7 × 10^4^ copies/mL). However, plasma and PBMC CMV DNA copy numbers remained low. Urine CMV DNA continued to be detected until the patient was 10 years of age (Fig. 2b). Although the hearing in her left ear remained normal during the 10-year follow-up period, hearing in the right ear deteriorated rapidly during the first 3 months but immediately returned to 60–70 dB and had not progressed at the 10-year follow-up (Fig. 2a).

**Fig. 3:**
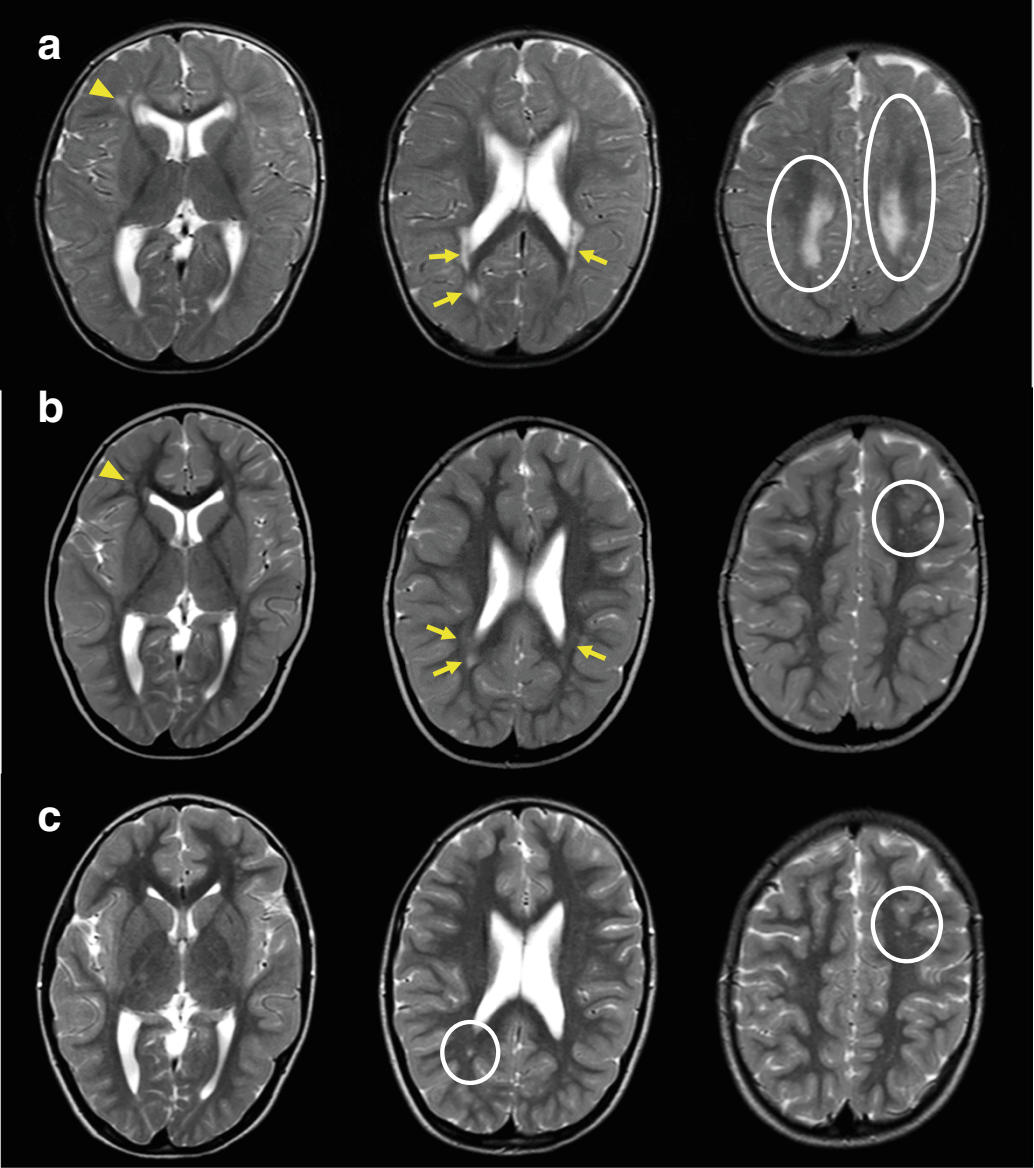
**a** Axial T2-weighted brain MRI. At 14 months of age (upper panels), abnormal areas of white matter were localized and remained around the lateral ventricle (arrow) and parietal lobes (round circles) in comparison to the findings at 1.5 months of age (see Fig. 1). The arrowhead indicates a periventricular cyst around the anterior horn of the right lateral ventricle. **b** At 3 years of age (middle panels), the abnormal white matter areas on T2-weighted images decreased further (arrow and circle). The periventricular cyst around the anterior horn of the right lateral ventricle was unchanged (arrowhead). **c** At 9 years of age (lower panels), a decreased volume of cerebral white matter was observed, but the abnormal areas of white matter had mostly disappeared, except for spotty signals in the parietal and occipital lobes (circles)

Follow-up brain MRI examinations were performed at 14 months, 3 years, and 9 years of age (Fig. 3a–c). The abnormal areas of white matter assessed on T2-weighted images were diffusely distributed in the occipital and parietal lobes at 1 month of age (Fig. 1) and were localized around the anterior and posterior horns of the bilateral lateral ventricles at 14 months of age (Fig. 3a). The white matter abnormalities decreased further at 3 years of age (Fig. 3b). At 9 years of age, the volume of cerebral white matter had decreased slightly, whereas the abnormal areas of white matter had mostly disappeared, except for spotty signals in the parietal and occipital lobes (Fig. 3c).

Regarding her clinical course, she showed no delays in motor milestones and was able to sit without support at 7 months and walk independently at 15 months. As of the most recent follow-up at 10 years of age, she is in 4th grade at a regular elementary school and can swim, dance, and even unicycle well. Her head circumference was within the normal range throughout the follow-up period Neurodevelopmental scores assessed by the Wechsler Scale for Children, Third edition (WISC-III) at 6 years of age and WISC-IV at 8 years of age were 93 and 103, respectively. At present, there is no evidence of balance disorders or emotional disturbances. The clinical course in this case is summarized in the supplemental time-line file (Additional file 1).

## Discussion and conclusions

We reported the case of a patient with cCMV infection who had severe neurological and hearing manifestations during infancy and was followed up for 10 years. At diagnosis, the typical radiological findings of cCMV infection include intracranial calcification, diffuse white matter abnormalities, and periventricular cysts. Several studies have demonstrated the relationship between the clinical manifestations of cCMV infection at birth and neurodevelopmental prognosis. Boppona et al. [5] reported a relationship between intracranial calcification during the neonatal period and poor long-term neurodevelopmental outcomes. Meanwhile, Inaba et al. [6] reported that an increased volume of white matter lesions on brain MRI was associated with a lower intelligence quotient. However, in the present case, the patient’s motor milestones and intelligence quotient were completely normal at 10 years of age despite radiological observations of intracranial calcification and severe white matter abnormalities during the early period of infancy.

SNHL is the most common sequela in cCMV infection. Hearing loss associated with symptomatic cCMV infection is often progressive (54% of patients) [7] and ultimately becomes severe to profound in the affected ear in 78% of patients [8]. Fortunately, in the present patient, the hearing in the affected right ear (60–70 dB) did not progress from baseline despite minimal fluctuations, and the hearing in the left ear remained normal at the 10-year follow-up (Fig. 2). Therefore, we speculate that the maintenance of hearing contributed to the improvement in her subsequent neurological and psychological development.

Some studies have reported that antiviral drugs have a beneficial effect on SNHL that starts during the neonatal period. Kimberlin et al. [9] reported that significantly fewer infants with SNHL who received GCV experienced worsening of hearing between baseline and ≥ 1 year than controls who did not received GCV (21% vs. 68%, respectively, *p* < 0.01). In a more recent study, patients who received oral VGCV for 6 months were more likely to show improvement in SNHL or retain normal hearing and had better neurodevelopmental scores including language-component and receptive-communication scales at 24 months than patients who received oral VGCV for 6 weeks [10]. Nevertheless, it is uncertain whether antiviral drugs are effective for preventing the progression of hearing loss in the long term. The two abovementioned studies focused on the short-term effects of antiviral drugs. Therefore, additional cases and long-term hearing observations are required to clarify the efficacy of antiviral drugs for SNHL.

In this patient, the dose of VGCV was lower than that used for conventional dosing (16 mg/kg/dose, twice daily) [10]. One reason for our dosing strategy is because the patient’s liver enzymes became elevated when intravenous GCV was administered at the recommended dose (6 mg/kg/dose, twice daily). The second reason is because the CMV viral load could be monitored frequently by quantitative PCR. It was therefore possible to determine the minimum dose needed to suppress the viral load. Fortunately, common adverse events such as neutropenia and thrombocytopenia were not observed during the treatment period.

In the present patient, the abnormal areas of white matter observed during infancy gradually localized at 3 years of age and mostly disappeared by 9 years. There is little information about the time-course of brain MRI findings in congenital CMV infections, including white matter abnormalities that occur with aging. MRI findings of nonprogressive or static white matter abnormalities have been described by van der Knaap et al. [11]. On the other hand, Krakar et al. [12] described the course of changing leukoencephalopathy in a case of symptomatic congenital CMV. They proposed that leukoencephalopathy was not only nonprogressive or static but also evolutive, which suggests both underlying disruption and delayed myelination. In our patient, MRI was repeatedly performed, which allowed evaluation of the delayed myelination and changes in white matter abnormalities, while the patient did not show any changes in status. Comparison of age-related changes in MRI images with developmental milestone provides important clinical information but is still controversial. Additional data may be required to resolve this issue.

Detection and quantification of CMV DNAemia may be helpful for predicting long-term adverse outcomes, particularly hearing loss [13]. Interestingly, in the present patient, bilateral hearing ability was maintained throughout 10 years of follow-up despite DNAemia during early infancy. Yamaguchi et al. [14] recently showed that newborns with both congenital CMV infection and SNHL had significantly higher urinary CMV DNA copy number than newborns with congenital CMV infection without SNHL (*p* = 0.036). In the present patient, urine CMV was detected continuously after 6 months of age until the 10-year follow-up, but viral load in the plasma and PBMC disappeared before 2 years of age. The clinical significance of reappearance of viral load in the plasma after treatment and/or PBMCs infected with CMV remain incompletely understood.

This study has several limitations. First, it is unknown whether antiviral therapy directly improved the symptoms associated with central nervous system involvement, including stabilizing bilateral hearing loss, reducing abnormal areas of white matter and preserving a normal IQ in this patient. The second limitation is the timing of antiviral drugs. GCV administration was started at 1.5 months of age, whereas conventional treatment should be started during the neonatal period [9, 10]. Therefore, further studies to determine the effectiveness of delayed administration of antiviral drugs would be of clinical interest. The third limitation is the indication for antiviral drugs of this patient. According to a report by Rawlinson et al. [15], neonates with mildly symptomatic congenital CMV infection should not be routinely given antiviral therapy. To data, only limited data have been collected regarding the long -term prognosis of cCMV infection. Further evaluation of a large number of these patients is necessary to clarify the natural course of cCMV infection and determine the safety and efficacy of antiviral therapy.

In conclusion, we reported the10-year follow-up case of cCMV in a patient who showed normal neurodevelopment, no progression of hearing loss, and ameliorating MRI findings, despite neurological complications including brain calcifications, severe white matter damage, periventricular cysts, and unilateral hearing loss during infancy.

## Additional file

Additional file 1: Time line picture. (DOCX 56 kb)

## Abbreviations

ABR: Auditory brainstem responce
ALT: Alanine aminotransferase
AST: Aspartate aminotransferase
cCMV: Congenital cytomegalovirus
CT: Computed tomography
DNA: Deoxyribonucleic acid
GCV: Ganciclovir
MRI: Magnetic resonance imaging
PBMC: Peripheral blood mononuclear cell
PCR: Polymerase chain reaction
SNHL: Sensorineural hearing loss
VGCV: Valganciclovir
WISC: Wechsler intelligence scale for children
