# Supplementary material for: 10-year follow-up of congenital cytomegalovirus infection complicated with severe neurological findings in infancy: a case report
Source: BMC Pediatr. 2018 Nov 23;18:369. doi: 10.1186/s12887-018-1348-8 (PMC6260854; doi:10.1186/s12887-018-1348-8)
Supplement: Supplementary file 1 — Time line picture. (DOCX 56 kb) [file 12887_2018_1348_MOESM1_ESM.docx]

Time line Picture

Introduced our hospital due to abnormalities of

Neonatal hearing screening at 1 month of age

**2007**

June

July

Aug

Oct

Nov

**2008**

**2009**

**2011**

Jan

**2012**

**2013**

**2014**

**2015**

**2016**

**2017**

**2018**

Diagnosis of congenital CMV

-Final Follow-up at 10 year-old

Mostly disappeared abnormal areas of white matter and maintained hearing ability (70, 20)

During antiviral therapy, right hearing loss transiently worsened to 90dB, but it returned to 60dB after treatment

VGCV 11mg/kg, twice daily (6wks)

GCV 6mg/kg, twice daily (5wks)

Decreased the abnormal area of white matter and Normal neurodevelopment at age of 3y

At 10 years of age, she showed normal neurodevelopment, no progression of hearing loss, and ameliorating MRI findings

Intellectual quotient (IQ): 103 (WISC-IV)

Intellectual quotient (IQ): 93 (WISC-III)

Right hearing loss (50dB)

Intracranial calcification (CT)

Diffuse white matter abnormality

(MRI)
